# Dexamethasone and Insulin Modulate Alanine Aminotransferase (ALT) Activity and Alanine Oxidation in C2C12 Cells in a Dose-Dependent Manner

**DOI:** 10.7759/cureus.59331

**Published:** 2024-04-30

**Authors:** Saed Woraikat, Defei Chen, Fuyu Yang, Chenglin Tang, Fan He, Kun Qian

**Affiliations:** 1 Department of Gastrointestinal Surgery, The First Affiliated Hospital of Chongqing Medical University, Chongqing, CHN; 2 Department of Gastrointestinal Surgery, The First Affiliated Hospital of Chongqing Medical University, Chongqing, TCD

**Keywords:** dexamethasone, insulin, expression, myocytes, alt, c2c12

## Abstract

Background: The muscle cells myocytes are differentiated for the purpose of contraction function, which plays a major role in body metabolism and energy haemostasis, through different metabolic pathways, such as glucose and protein metabolic pathways. Alanine aminotransferase (ALT) plays a crucial role by reversibly catalysing transamination between alanine and a-ketoglutarate to form pyruvate and glutamate and by mediating the conversion of these four major intermediate metabolites. ALT plays important roles for energy homeostasis during fasting and prolonged exercise anaerobically, when muscle protein must first be broken down into its constituent amino acids.

Methods: Mouse skeletal myoblast cell line C2C12 was cultured in Dulbecco's modified eagle medium (DMEM) growth medium, supplied with 2% horse serum supplemented with 1 uM insulin, 2 mM glutamine and penicillin and streptomycin antibiotics for seven days. The differentiation medium is refreshed every 24 hours. Then, C2C12 cells were treated with insulin and dexamethasone to examine their effects on myocytes' ALT activity.

Results: In our study, we found an impact on ALT activity under different influences, including C2C12 differentiation, dexamethasone and insulin treatments, which shed light on the dynamic interplay between ALT activity, alanine metabolism, and cellular states, like differentiation and stress responses.

Conclusion: The study provides valuable insights into the dynamic regulation of ALT activity and alanine metabolism in C2C12 cells across differentiation and drug treatments. Further research is encouraged to explore the underlying mechanisms and their implications for muscle function, differentiation and potential therapeutic interventions in metabolic disorders.

## Introduction

Myocytes are highly specialized cells that play a vital role in the muscle fiber's ability to produce tension during contraction, generating force that aids in physical activity and maintaining cellular energy balance. The C2C12 cell line has the ability to differentiate into multinucleated myotubes, making it a valuable tool for investigating various aspects of muscle biology, including gene expression and signaling pathways. Furthermore, it represents an immortalized lineage of murine skeletal myoblasts, initially isolated from the satellite cells of the thigh muscle [1]. The enzyme alanine aminotransferase (ALT) is involved in the process of transferring an amino group between alanine and α-ketoglutarate, leading to the production of pyruvate and glutamate [2]. This plays a crucial role in the metabolism of glucose and proteins. ALT plays a vital role in maintaining energy balance during periods of fasting and intense anaerobic exercise. It helps in converting four important intermediate metabolites. This process is essential for the breakdown of muscle protein into amino acids, which are subsequently utilised to fulfil the increasing requirement for adenosine triphosphate (ATP) production. During exercise, there is a notable rise in the rate of glycolysis, resulting in a surplus of pyruvate for the alanine transaminase (ALT) process.

Certain experts have emphasised the significance of the ALT mechanism, as it allows for the creation of alanine from pyruvate. This process leads to the export of nitrogen as alanine and the restoration of α-ketoglutarate [3]. When muscle blood is released, it contains a significant amount of alanine and glutamine. These two amino acids make up a substantial portion, up to 30%, of the total amino acids released by the muscle [4]. This upregulation contributes to maintaining cellular energy balance and ensuring the continued ability of myocytes to contract effectively. ALT forms connections with other metabolic enzymes in myocytes, creating a complex system of interconnected pathways that impact the production and utilisation of energy. ALT also plays a role in the detoxification of ammonia, which is a byproduct of protein breakdown, and as mentioned in previous studies ammonia impairs protein synthesis in myocytes [5,6].

Accumulation of ammonia in muscle tissues can impede the proper functioning of muscle cells. Furthermore, ALT effectively converts ammonia into alanine. The liver takes up alanine produced from muscle and converts it to pyruvate through the ALT enzyme. The pyruvate is subsequently utilised in the production of glucose via gluconeogenesis [7]. Alanine has the ability to be converted directly to pyruvate and can be oxidised through the tricarboxylic acid cycle (TCA) to serve as a source of energy for muscles during extended periods of starvation and anaerobic activity after muscle glycogen stores have been used up. Thus, ALT plays a critical role in providing the necessary carbon source for gluconeogenesis and the oxidation of amino acids. These processes are essential for the survival of animals during times of fasting and anaerobic activity.

Given the significance of ALT in the oxidation of ammonia acid in muscle, we utilised ALT isoform-specific antibodies to evaluate the expression, regulation and protein levels of ALT1 and ALT2. In addition, we explored their potential role in the oxidation of alanine in myocytes. Obtaining a deep understanding of this intricate connection offers valuable insights into the mechanisms that support the functioning of myocytes and may uncover potential treatment targets for disorders affecting myocyte energy metabolism.

## Materials and methods

Cell culture and treatment

C3H mouse skeletal myoblast cell line C2C12, 6 replicates per treatment group were cultured in a growth medium of Dulbecco's modified eagle medium (DMEM) (Invitrogen, Grand Island, NY) containing 20% fetal bovine serum (Invitrogen), 2 mM glutamine, penicillin and streptomycin antibiotics (Cellgro, Manassas, VA) for passage. For myocyte differentiation, fully confluent cells were maintained in DMEM supplied with 2% horse serum supplemented with 1 uM insulin, 2 mM glutamine and penicillin and streptomycin antibiotics for seven days. In addition, the cells were incubated in an atmosphere with 8% CO_2_, maintained at 37°C with controlled humidity, and the differentiation medium was refreshed every 24 hours.

ALT activity assay

The alanine aminotransferase kit (Calchem, Huntington, NY) was used to measure ALT activity, following the instructions provided by the manufacturer. A cell lysate sample (10 µL) was combined with a mixture of reagents A and B (200 µL), which included L-alanine, LDH, NADH and 2-oxoglutarate, and incubated at a temperature of 25°C. The absorbance at a wavelength of 340 nm was measured for a duration of five minutes, with readings taken every 30 seconds following the introduction of protein. The protein concentration of cell lysates was used to correct the final ALT activities. The ALT activity unit was determined as the quantity of enzyme needed to produce 1 mol/L of NAD per minute under the specified assay conditions at a temperature of 25°C.

Western blot analysis

C2C12 mouse cells were homogenised and lysed in a lysis buffer containing 50 mM HEPES, 1 mM EDTA, 150 mM NaCl, 1% Triton X 100 and a cocktail of proteinase inhibitors (Sigma). Lysates containing 20 µg of protein were separated in 7.5% SDS-PAGE (sodium dodecyl sulfate-polyacrylamide gel electrophoresis) gel and transferred to polyvinylidene difluoride membranes (Millipore Corporation, Billerica, MA). The membranes were then incubated with affinity-purified, biotin-labelled rabbit anti-ALT1 or rabbit anti-ALT2 polyclonal antibody in TTBS buffer with 5% milk at 1:10,000 dilution at 4°C overnight. Streptavidin-conjugated alkaline phosphatase (Thermo Scientific, Rockford, IL) at a 1:5000 dilution was used for detection. The blots were visualised by a BCIP/NBT (5-bromo-4-chloro-3-indolyl phosphate/nitro blue tetrazolium) Western blot kit (Sigma) and quantified by measuring optical density values with ImageJ software.

Statistics

Results are expressed as mean + standard error (SE). A student's t-test was used to inspect the differences between means (GraphPad Software, San Diego, CA). Differences at a P-value of 0.05 were considered statistically significant.

## Results

ALT activity in C2C12 differentiation

**Figure 1:**
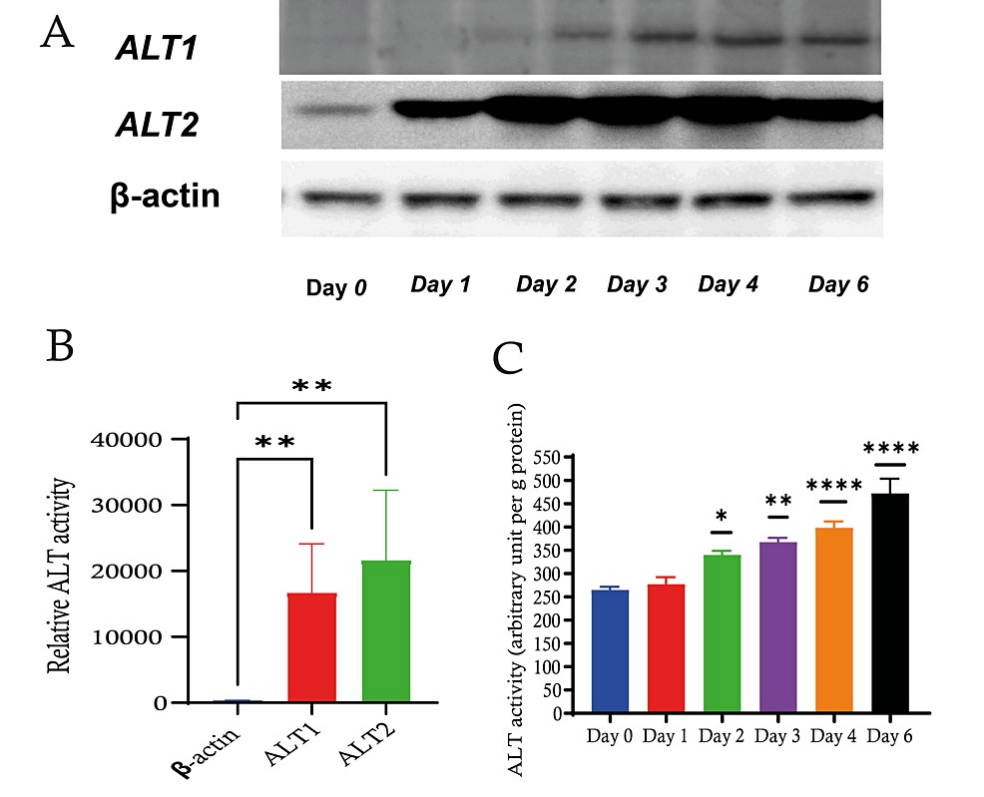
ALT activity during C2C12 differentiation A) Western blot representing the expression levels of ALT1 and ALT2, where β-actin serves as a loading control. B) Relative ALT activity compared to the control group. C) Quantitative analysis depicting the activity of ALT over a six-day period suggests an increase in ALT activity from day 0 to day 6, peaking on day 6. Data are expressed as mean ± S.E. * p < 0.05; **: p < 0.01; ****: p < 0.0001. ALT: alanine aminotransferase, SE: standard error.

We investigated the enzymatic activity of alanine aminotransferase (ALT) in C2C12 mouse myoblasts for a duration of six days. The measurement of ALT activity was conducted in arbitrary units per gramme of protein in order to assess the cellular response during myoblast development. During a six-day period, we observed an increase in ALT activity, reaching its highest point on the sixth day (Figure 1). The gradual elevation in ALT activity seen in C2C12 cells indicates dynamic cellular activity taking place over a period of six days, which shows that there could be a correlation between the increase in ALT activity and the differentiation into myotubes. This process might lead to a boost in amino acid metabolism and protein synthesis, showing increased metabolic activity that may indicate different stages of cell differentiation.

C2C12 treated with dexamethasone and insulin

**Figure 2:**
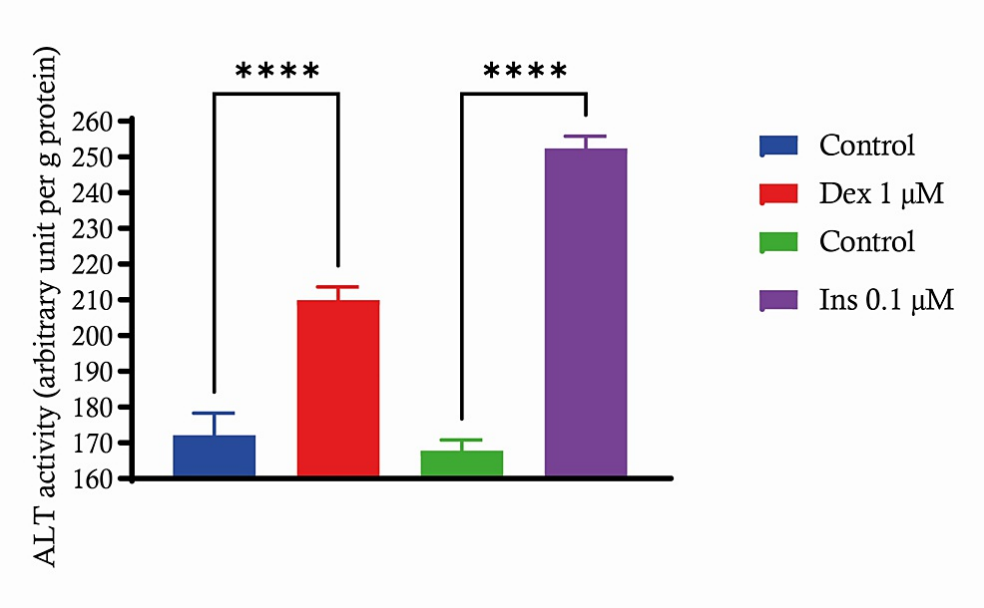
Insulin and dexamethasone effects on ALT activity in C2C12 cells Demonstrating ALT activity changes under the influence of insulin 0.1 μM and dexamethasone 1 μM treatments. Data are expressed as mean ± S.E. ****: p < 0.0001. ALT: alanine aminotransferase, SE: standard error.

C2C12 cells were subjected to a concentration of 1 μM dexamethasone (Dex) or 0.1 μM insulin (Ins) in order to examine the activity of ALT in C2C12 cells. The C2C12 cells, when exposed to 1 μM dexamethasone, exhibited a significant increase in ALT activity (P < 0.0001) in comparison to the control group. This indicates that dexamethasone treatment enhances the activity of the ALT enzyme. Our findings indicate that treating C2C12 with 0.1 μM insulin resulted in a greater and more significant increase in ALT activity (P < 0.0001) compared to the control. This suggests that insulin has a strong stimulating impact on ALT activity at the given concentration. The findings indicate that both dexamethasone and insulin noticeably increase ALT activity in C2C12 cells at the tested doses. The effects of insulin are particularly remarkable, which is crucial to comprehending insulin's function in muscle cell metabolism. The rise in ALT activity might be indicative of modifications in cellular metabolism as a result of these treatments, potentially indicating altered amino acid catabolism or anaplerosis in these myoblasts (Figure 2).

C2C12 treated with high-dose dexamethasone (1 µM) and low-dose dexamethasone (0.1 µM)

**Figure 3:**
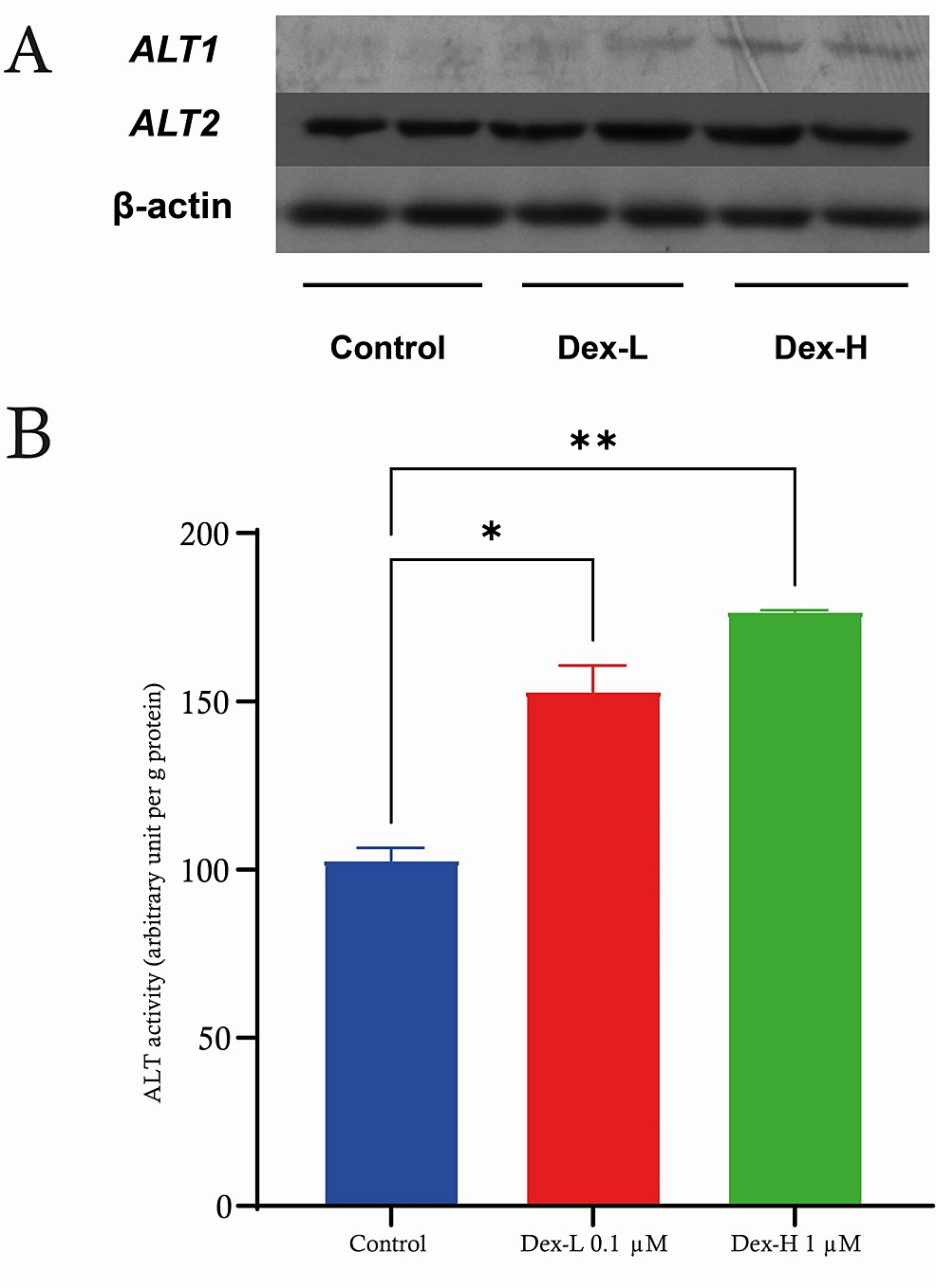
C2C12 treated with high-dose dexamethasone (1 µM) and low-dose dexamethasone (0.1 µM) A) Indicating the activity of ALT in C2C12 cells treated with two concentrations of dexamethasone during a period of 72 hours compared to untreated control cells. B) Western blot analysis of ALT1 and ALT2 protein expression in C2C12 cells following treatment with dexamethasone (1 µM and 0.1 µM) in comparison to control. β-actin is used as a loading control. Data are expressed as mean ± S.E. * p < 0.05; **: p < 0.01. ALT: alanine aminotransferase, SE: standard error.

The C2C12 cells were subjected to dexamethasone at doses of 0.1 µM (Dex-L) and 1 µM (Dex-H). We established a control group as our baseline, where no dexamethasone medication was administered. Our observation revealed that the groups treated with Dex-L had a significant increase in ALT activity compared to the control group (p < 0.05). Furthermore, we observed a significant increase in ALT activity (P < 0.01) in the cells treated with Dex-H compared to both the control group and Dex-L group. This shows that dexamethasone influences the activity of ALT in C2C12 cells in a manner that is dependent on its concentration (Figure 3).

Alanine oxidation in C2C12 treated with dexamethasone (1 µM), insulin (0.1 µM) and D-cycloserine

**Figure 4:**
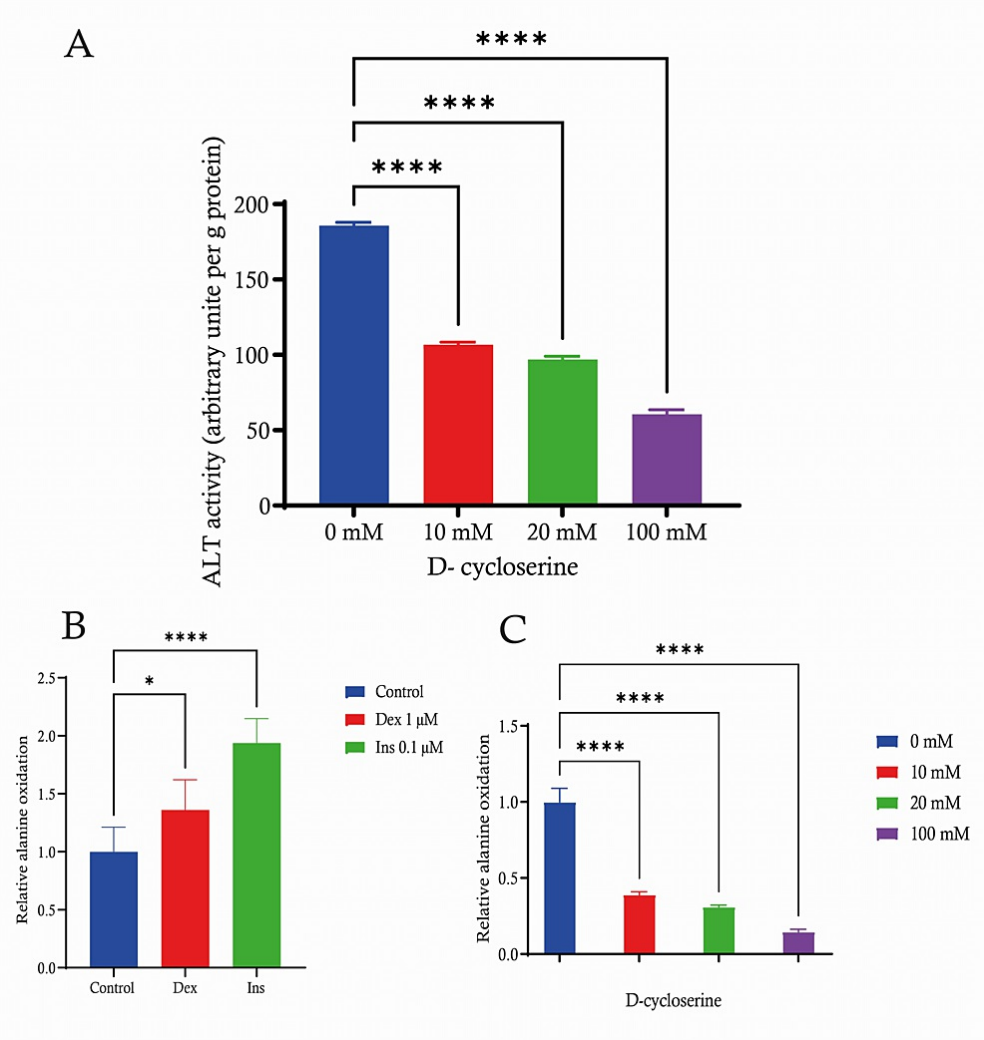
Alanine oxidation in C2C12 treated with dexamethasone, insulin and D-cycloserine A) Quantitative analysis representing the activity of ALT in C2C12 cells under various concentrations of d-cycloserine. B) representing the alanine oxidation in C2C12 cells treated with dexamethasone (Dex) or insulin (Ins) compared to the untreated control. C) Illustrating the effect of different concentrations of d-cycloserine on alanine oxidation in C2C12 cells. Data are expressed as mean ± S.E. * p < 0.05; ****: p < 0.0001. ALT: alanine aminotransferase, SE: standard error.

C2C12 cells were studied using different concentrations of D-cycloserine. We performed measurements in C2C12 cells using different doses of D-cycloserine. When comparing the control group with a concentration of 0 mM D-cycloserine, we found that ALT activity decreased in a dose-dependent manner after treatment with D-cycloserine. Cells subjected to a concentration of 10 mM D-cycloserine revealed a significant decrease in enzyme activity in comparison to the control group. Even with the 20 mM concentration, there was a further reduction in ALT activity. When testing at the maximum dose of 100 mM D-cycloserine, the activity of ALT was significantly decreased compared to all other groups, including the values of 10 and 20 mM. The user's text is empty. Our results demonstrate that D-cycloserine exhibits an inhibitory effect on ALT activity in C2C12 cells, as evidenced by a statistically significant decrease (p < 0.0001) at various doses. Moreover, higher concentrations of D-cycloserine result in greater degrees of suppression (Figure 4A). We investigated the impact of 1 µM dexamethasone (Dex) and 0.1 µM insulin (Ins) on the relative alanine oxidation in C2C12 cells. The control group, which does not receive any therapy, serves as a reference point for comparison and is set to a value of 1 for the purpose of measuring relative alanine oxidation.

Administration of 1 µM Dex led to a significant increase in alanine oxidation (p < 0.05) when compared to the control group. By contrast, cells exposed to a concentration of 0.1 µM of insulin exhibited statistically significant increase (p < 0.0001) in the relative alanine oxidation rate when compared to the control group. Insulin at a concentration of 0.1 µM significantly elevates the oxidation of alanine in C2C12 cells compared to both the control and 1 µM Dex dose (Figure 4B). The relative alanine oxidation in C2C12 cells was evaluated after exposure to different concentrations of D-cycloserine (0 mM, 10 mM, 20 mM and 100 mM). The control group, which was not exposed to D-cycloserine, had the most significant level of alanine oxidation activity. This activity was standardised to a value of about 1.0 for the purpose of this research. We observed a dose-dependent alanine oxidation decrease. An inverse relationship between the dosage and the rate of alanine oxidation is illustrated. The relative alanine oxidation was markedly decreased (p < 0.0001) compared to the control at a dose of 10 mM. The inhibition observed was significantly more effective at a dose of 20 mM (p < 0.0001), suggesting a further decline in the activity of alanine oxidation. The greatest dose of D-cycloserine (100 mM) resulted in a significant drop in alanine oxidation, with activity levels approaching the baseline. These findings may have an impact on understanding the metabolic impacts of D-cycloserine on muscle cells (Figure 4C).

Alanine oxidation during C2C12 differentiation

**Figure 5:**
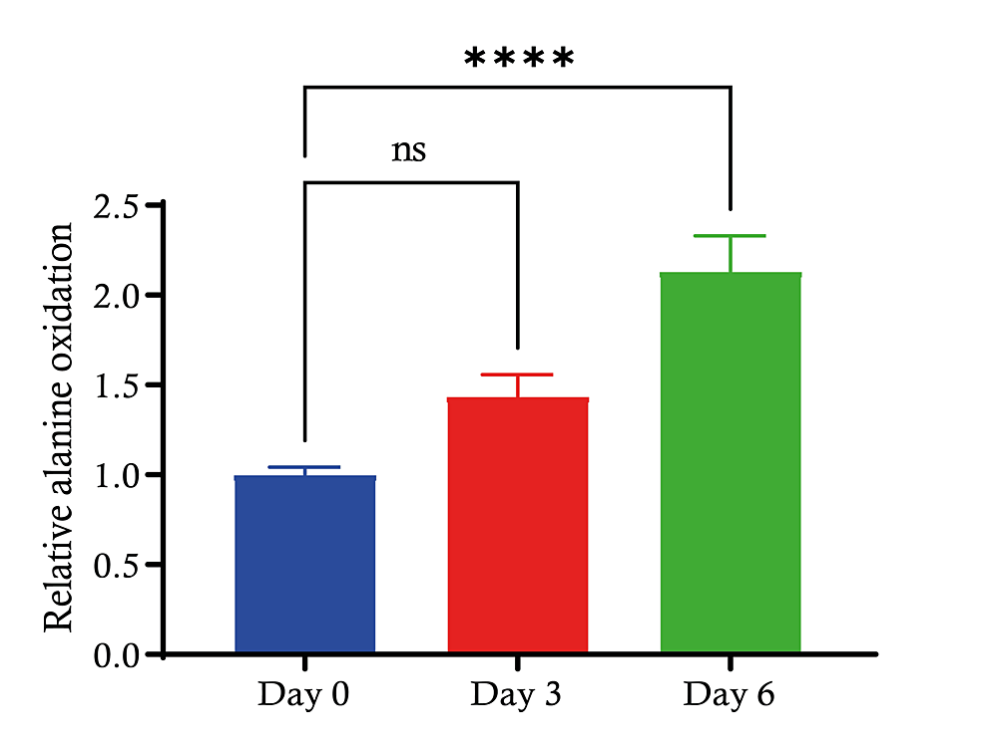
Alanine oxidation during C2C12 differentiation Bar graph showing the relative alanine oxidation in C2C12 myoblasts at three different time points: initial (day 0), mid-differentiation (day 3) and late differentiation (day 6). The relative oxidation is normalised to the initial value on day 0. Data are expressed as mean ± S.E. ****: p < 0.0001. SE: standard error.

In our experiment, we measured the relative alanine oxidation in C2C12 cells at three different time points: day 0, day 3 and day 6. On day 0, the relative alanine oxidation level served as the baseline reference for the experiment. By day 3, there was a non-significant observable increase in relative alanine oxidation compared to day 0. On day 6, the relative alanine oxidation appears to have increased further compared to day 3. The increase is statistically significant (p < 0.0001) when compared to the baseline level on day 0. These findings might suggest a time-dependent metabolic adaptation or cellular change that affects alanine oxidation (Figure 5).

Alanine oxidation of different doses of alanine treatment

**Figure 6:**
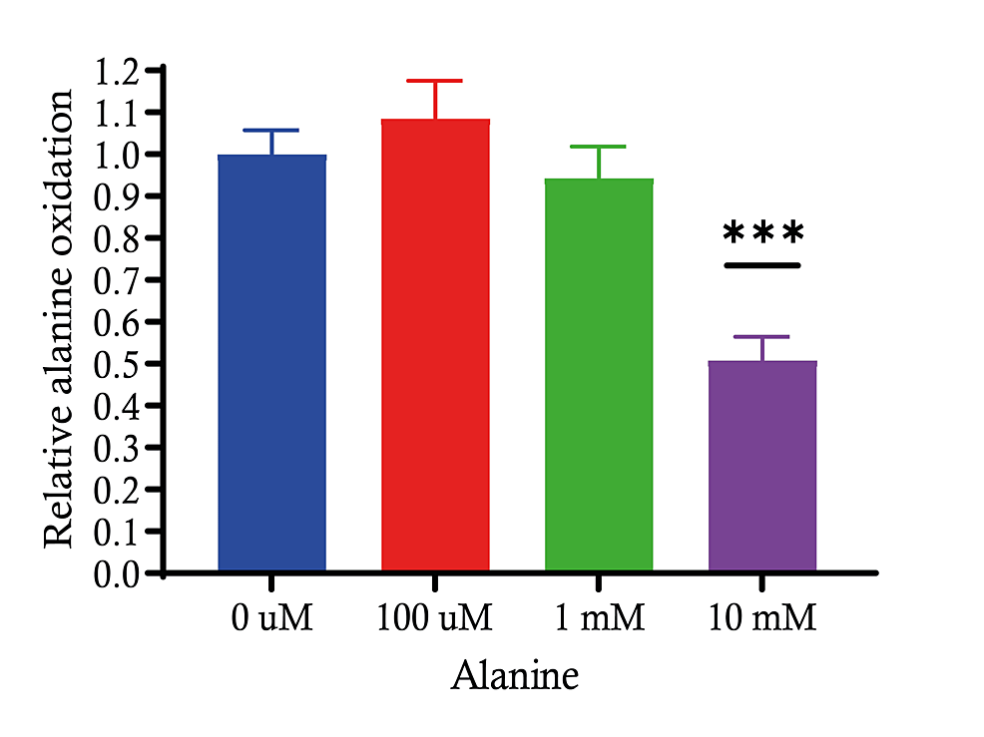
Alanine oxidation of different doses of alanine treatment The graph depicts the relative alanine oxidation in response to different concentrations of alanine treatment. The oxidation is shown relative to the untreated control. Data are expressed as mean ± S.E. *** p < 0.001. SE: standard error.

Studying the relative alanine oxidation in C2C12 cells at varying concentrations of alanine, we aim to investigate the effects of different concentrations of alanine on the relative oxidation of the same amino acid in C2C12 cells. Establishing the baseline relative alanine oxidation at the control concentration (0 µM), against which the effects of higher alanine concentrations were measured. Treatment with 100 µM alanine did not result in a statistically significant change in relative alanine oxidation as compared to the control. An increase in alanine concentration to 1 mM also did not show a significant difference in oxidation levels when compared to the control or the 100 µM concentration. However, when the alanine concentration was increased to 10 mM, there was a statistically significant decrease in relative alanine oxidation (p < 0.001). In conclusion, these results suggest that while low to moderate increases in alanine concentration (up to 1 mM) do not significantly affect its relative oxidation in C2C12 cells, a high concentration (10 mM) inhibits the oxidation process significantly, which may imply a saturation effect or a feedback inhibition mechanism at higher concentrations of alanine (Figure 6).

Alanine oxidation of ALT overexpression

**Figure 7:**
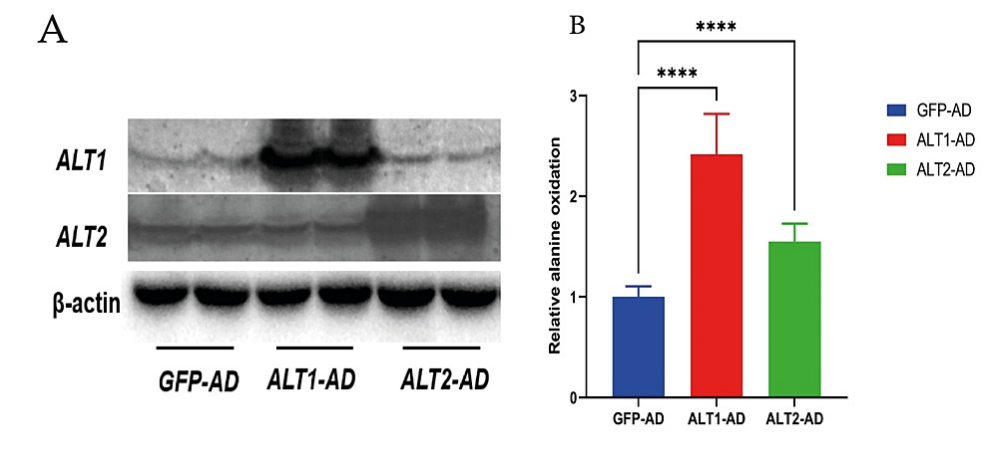
Alanine oxidation of ALT overexpression A) Western blot displaying the protein expression levels of ALT1 and ALT2 following transfection with their respective overexpression constructs (ALT1-AD and ALT2-AD) compared to a control vector (green fluorescent protein-adenovirus (GFP-AD)). β-actin is used as a loading control. B) Quantitative analysis of relative alanine oxidation in cells overexpressing either ALT1-AD or ALT2-AD, normalised to the control GFP overexpression. Data are expressed as mean ± S.E. ****: p < 0.0001. ALT: alanine aminotransferase, SE: standard error.

We measured the relative alanine oxidation in C2C12 cells expressing different alanine transporters: GFP-AD (control), ALT1-AD and ALT2-AD. The control cells were transfected with a green fluorescent protein (GFP) to monitor transfection efficiency, while the other two sets of cells were transfected with constructs for alanine transporter 1 (ALT1) and alanine transporter 2 (ALT2), respectively. Upon expression of ALT1-AD, there was a significant increase in relative alanine oxidation (p < 0.0001), more than doubling the baseline level observed in GFP-AD cells. The cells expressing ALT2-AD also showed a significant increase in relative alanine oxidation (p < 0.0001) compared to the GFP-AD control, but this increase was less pronounced than that observed with ALT1-AD. This demonstrates that the overexpression of ALT1 significantly enhances alanine oxidation in C2C12 cells, suggesting that ALT1 may be more efficient or have a higher capacity for transporting alanine compared to ALT2. The increase in alanine oxidation in cells expressing ALT2-AD also suggests a role for this transporter in alanine metabolism, although its effect is less pronounced than that of ALT1 (Figure 7).

## Discussion

Our study explores the dynamic behaviour of ALT activity and alanine oxidation in C2C12 myoblast cells, revealing metabolic adaptations during differentiation, and exposure to various treatments, including dexamethasone and insulin. ALT involves a reversible transamination reaction, catalysing the interconversion of alanine and α-ketoglutarate to form pyruvate and glutamate. Occurring through transamination in which ALT catalyses the transfer of an amino group from alanine to the α-ketoglutarate, resulting in the formation of pyruvate and glutamate. Then, there is a reverse transamination, which allows for the conversion of pyruvate back to alanine [2]. ALT has a role in different pathways and cycles, such as the alanine-glucose cycle facilitating the interconversion of alanine and glucose between muscle cells and the liver, supporting glycogen replenishment and muscle energy requirements via gluconeogenesis [8]. It also plays a role in glutamate-pyruvate cycle, during physical activity or a starvation state, muscle tissue breaks down proteins, releasing glutamate, which then undergoes transamination with pyruvate by ALT, forming alanine, which then is transported to the liver via the bloodstream. Subsequently, ALT again converts alanine back to pyruvate, which can be used for energy production via gluconeogenesis [9]. Furthermore, although ALT does not directly engage in the urea cycle, its pivotal function in pyruvate production provides essential support to this metabolic pathway [10]. The process of C2C12 myoblast differentiation is characterised by a sequence of events encompassing myoblast proliferation, elongation and subsequent fusion, occurring across multiple stages under tight regulatory control [11].

In our study findings, we found that ALT activity increases during differentiation. During the six-day differentiation of C2C12 cells, there is a noticeable and continuous rise in ALT activity. This indicates a dynamic cellular process that occurs alongside myoblast differentiation. The spike in ALT activity on day six suggests a boost in metabolic activity and heightened amino acid metabolism linked to the development of myotubes. We predict that the increase in ALT activity is most likely due to the shifting metabolic needs of differentiating cells, which involves an increase in protein synthesis. The significance of ALT in cellular differentiation and its potential contribution to muscle tissue development is highlighted by these findings, which suggest progressive upregulation of alanine metabolism as cells differentiate into mature myocytes. It is worth noting that during periods of fasting, there was an observed increase in ALT2 mRNA levels in both skeletal muscle and liver [12]. In a study examining the effects of prolonged fasting, researchers observed a significant decrease in circulating FFAs, which was likely caused by the presence of high insulin levels. Interestingly, the subjects still exhibited slightly higher rates of gluconeogenesis from anaplerosis [13]. These findings suggest that the increased ALT activity indicates accelerated amino acid mobilisation and catabolism for energy under nutrient scarcity.

Upon examining the responsiveness of ALT to dexamethasone and insulin, we observed an increase in ALT activity in C2C12 cells. Prior studies have indicated that administering dexamethasone (Dex) during the myotube maturation phase elicits a dose-dependent atrophic response in myotubes [14-16]. During our study, we found that exposure to dexamethasone has shown an enhancement of ALT enzyme activity, which varies based on concentration and emphasises the significance of proper dosing when evaluating the impact of dexamethasone. At lower concentrations, there is a moderate increase in ALT activity, whereas at higher concentrations, we found the effect to be more pronounced. The variation in ALT activity influenced by dexamethasone at different concentrations indicates that varying doses can have specific impacts on amino acid metabolism and cellular functions in myoblasts, which may indicate changes in amino acid catabolism or anaplerosis as a response to dexamethasone. By contrast, insulin has shown a strong stimulating effect on ALT activity, indicating its crucial role in controlling muscle cell metabolism. These observations show that both dexamethasone and insulin play pivotal roles in regulating muscle amino acid metabolism, potentially promoting protein synthesis. This highlights the intricate nature of dexamethasone and insulin regulation in muscle cells and its influence on amino acid metabolism. Elevated ALT levels are commonly observed in inflammatory muscle diseases, like polymyositis and dermatomyositis, as well as in conditions, such as rhabdomyolysis, characterised by muscle weakness and damage. This suggests a potential link between ALT and muscle injury, although the underlying mechanisms remain unclear. Given ALT's involvement in amino acid metabolism and energy production, it likely plays critical roles in maintaining muscle health and repair. This study aims to comprehensively understand the changes in ALT levels during muscle disorders, potentially identifying new therapeutic targets for improving clinical management strategies [17,18].

We hypothesised that D-cycloserine might alter cellular metabolism and physiology by affecting ALT activity, thereby impacting downstream effects on amino acid balance and energy production, considering its ability to inhibit the activity of alanine racemase, which influences the balance of L-alanine and D-alanine [19,20]. Interestingly, studying the impact of D-cycloserine on ALT activity and alanine oxidation in C2C12 cells at different concentrations presents a significant inhibition effect on ALT activity and alanine oxidation, in which the higher the concentration, the stronger the inhibition effects. Implementing the idea that D-cycloserine might interfere with alanine metabolism through the direct inhibition of ALT, and alanine oxidation indicates its potential involvement in the regulation of amino acid metabolism pathways.

Investigating the alanine oxidation during C2C12 cell differentiation, the increase in alanine oxidation over time suggests that differentiation of C2C12 cells is associated with an upregulation of metabolic processes involving alanine. This could reflect the increased demand for energy and biosynthetic precursors necessary for the growth and development of differentiating muscle cells.

Alanine, derived from the transamination of glutamate with pyruvate, is transported from the muscle to the liver for conversion into glucose [21]. By studying the alanine oxidation of different doses of alanine treatment, we found that low to moderate increases in alanine concentration do not have a significant impact on its relative oxidation. Interestingly, when the concentration of alanine is high (10 mM), it significantly inhibits the oxidation process. This suggests that high concentrations of alanine may inhibit the oxidation process, possibly due to substrate inhibition, metabolic feedback mechanisms or toxicity at high substrate concentrations. ALT1 and ALT2 have distinct tissue distributions. The expression of ALT1 is significantly elevated in the colon, intestine, adipose tissue and liver. The expression of ALT2 is elevated in the brain, adipose tissue, liver and skeletal muscle [22]. Studying alanine oxidation of ALT overexpression of both forms of ALT leads to increased alanine oxidation, with ALT1 having a more pronounced effect than ALT2. This suggests that ALT1 is more effective in catalysing the chemical reactions involved in alanine oxidation. ALT enables muscles to transfer nitrogen to the liver through the continuous cycling of organic acids. The ALT pathway relies on a continuous source of pyruvate as an amino acceptor [23], providing valuable insights into the precise roles of these transporters in controlling amino acid metabolism.

Study’s limitations

This study explores the regulation of ALT activity in C2C12 murine myoblast cells, highlighting the limitations of the in vitro model in replicating in vivo metabolic complexities. The research focuses on ALT activity and its modulation by various factors but acknowledges that alanine metabolism involves multiple enzymes and pathways. The findings reveal dose-dependent responses to insulin, dexamethasone and D-cycloserine, but caution is needed as the concentrations used may not reflect physiological levels. The study also acknowledges the influence of specific culture conditions on cellular metabolism and ALT activity, which could affect the interpretation and translational relevance of the findings. The study suggests potential alternative mechanisms, but its translational relevance to human physiology and clinical conditions remains uncertain. Further validation through animal models and clinical studies is necessary to confirm findings and explore potential implications for metabolic disorders and therapeutic strategies.

## Conclusions

Our study reveals the dynamic changes in ALT activity and alanine oxidation during C2C12 myoblast differentiation and exposure to treatments like dexamethasone, insulin and D-cycloserine. These findings underscore the intricate regulatory mechanisms governing amino acid metabolism in muscle cells. They also highlight the potential significance of ALT in cellular differentiation and muscle myogenesis which has a role in the growth, repair and regeneration of muscle tissues. Furthermore, our observations shed light on the differential effects of dexamethasone, insulin and D-cycloserine on ALT activity, emphasising their roles in modulating muscle cell metabolism and suggesting potential avenues for further research in the field.
